# Effect of Essential Oils on the Release of TNF-α and CCL2 by LPS-Stimulated THP‑1 Cells

**DOI:** 10.3390/plants10010050

**Published:** 2020-12-28

**Authors:** Maria Graça Miguel, Carina Isabel da Silva, Luana Farah, Fernão Castro Braga, Ana Cristina Figueiredo

**Affiliations:** 1 Mediterranean Institute for Agriculture, Environment and Development (MED), Departamento de Química e Farmácia, Faculdade de Ciências e Tecnologia, Universidade do Algarve, Campus de Gambelas, 8005-139 Faro, Portugal; carina.isabel.silva@hotmail.com; 2 Departamento de Produtos Farmacêuticos, Faculdade de Farmácia, Universidade Federal de Minas Gerais, Av. Antônio Carlos 6627, Pampulha, 31.270-901 Belo Horizonte, Brazil; luanafarah@hotmail.com (L.F.); fernaobraga@farmacia.ufmg.br (F.C.B.); 3 Centro de Estudos do Ambiente e do Mar (CESAM Lisboa), Faculdade de Ciências da Universidade de Lisboa, Centro de Biotecnologia Vegetal (CBV), DBV, C2, Piso 1, Campo Grande, 1749-016 Lisboa, Portugal; acsf@fc.ul.pt

**Keywords:** essential oils, cytokine, chemokine, inflammation

## Abstract

Plants and their constituents have been used to treat diverse ailments since time immemorial. Many plants are used in diverse external and internal formulations (infusions, alcoholic extracts, essential oils (EOs), etc.) in the treatment of inflammation-associated diseases, such as those affecting the respiratory tract or causing gastrointestinal or joint problems, among others. To support the traditional uses of plant extracts, EOs have been assessed for their alleged anti-inflammatory properties. However, the effect of EOs on the release of cytokines and chemokines has been much less reported. Considering their traditional use and commercial relevance in Portugal and Angola, this study evaluated the effect of EOs on the in vitro inhibition of the cytokine tumor necrosis factor-α (TNF-α) and the chemokine (C-C motif) ligand 2 (CCL2) by lipopolysaccharide (LPS)-stimulated human acute monocytic leukemia cells (THP-1 cells). Twenty EOs extracted from eighteen species from seven families, namely from Amaranthaceae (*Dysphania ambrosioides*), Apiaceae (*Foeniculum vulgare*), Asteraceae (*Brachylaena huillensis*, *Solidago virgaurea*), Euphorbiaceae (*Spirostachys africana*), Lamiaceae (*Lavandula luisieri*, *Mentha cervina*, *Origanum majorana*, *Satureja montana*, *Thymbra capitata*, *Thymus mastichina*, *Thymus vulgaris*, *Thymus zygis* subsp. *zygis*), Myrtaceae (*Eucalyptus globulus* subsp. *maidenii*, *Eucalyptus radiata*, *Eucalyptus viminalis*) and Pinaceae (*Pinus pinaster*) were assayed for the release of CCL2 and TNF-α by LPS-stimulated THP-1 cells. *B. huillensis, S. africana, S. montana, Th. mastichina* and *Th. vulgaris* EOs showed toxicity to THP-1 cells, at the lowest concentration tested (10 μg/mL), using the tetrazolium dye assay. The most active EOs in reducing TNF-α release by LPS-stimulated THP-1 cells were those of *T. capitata* (51% inhibition at 20 μg/mL) and *L. luisieri* (15–23% inhibition at 30 μg/mL and 78–83% inhibition at 90 μg/mL). *L. luisieri* EO induced a concentration-dependent inhibition of CCL2 release by LPS‑stimulated THP-1 cells (23%, 54% and 82% inhibition at 10, 30 and 90 μg/mL, respectively). These EOs are potentially useful in the management of inflammatory diseases mediated by CCL2 and TNF‑α, such as atherosclerosis and arthritis.

## 1. Introduction

Since ancient times, man has used the plant kingdom as a source for clothing, construction, fuel, food, spices and medicines, as well as for poisons. Nowadays, around half the pharmaceutical drugs used in developed countries, such as aspirin, are of plant origin [1]. Traditional medicine is still the main source of health care for 80% of the people in developing countries, where medicinal plants are commonly used for the treatment of several ailments, notably inflammatory diseases. 

Acute inflammation is a short-term reaction which is essential for survival after an infection or a physical injury. On the other hand, chronic inflammation, promoted by social, environmental and lifestyle factors (diet, smoking, alcoholism, inactivity), may trigger diverse long-term illnesses such as cardiovascular disease, cancer, diabetes mellitus, chronic kidney disease, nonalcoholic fatty liver disease and autoimmune and neurodegenerative disorders [2]. These diseases and lifestyles are associated with atherosclerosis, the early detection of which is based on peripheral artery and carotid artery thickness. In a recent study of the epidemiological burden caused by carotid atherosclerosis, Song et al. [3] estimated that, in 2020, the prevalence of increased carotid intima-media thickness in people aged 30 to 79 years was about 28%, equivalent to approximately 1070 million cases worldwide.

In Portugal, recent studies showed that as many as 740,000 adults are affected by atherosclerosis [4]. Data on other inflammatory diseases in Portugal, such as rheumatic diseases [5], showed that women (64%) are more affected by rheumatic diseases, including rheumatoid arthritis, than men (47%). These diseases are underdiagnosed in Portugal and are responsible for disability and absenteeism at work, with the consequent individual, social and economic costs [5]. Much less is known about inflammatory diseases in other Portuguese-speaking countries. There are no studies on the prevalence of rheumatic diseases in Angola [6], although the occurrence of rheumatic fever, rheumatoid arthritis or systemic lupus erythematosus is known [7].

Acute inflammation can be treated by using oral nonsteroidal anti-inflammatory drugs (NSAIDs), despite being associated with adverse gastrointestinal and cardiovascular effects [8,9]. It would be desirable to identify natural plant products with anti-inflammatory properties but with fewer adverse effects. Despite the traditional application of plants, their effects have not always been proven by scientific evidence. On the other hand, scientific research sometimes provides evidence of biological activities for which the plants in question had never been traditionally used.

Plant extracts, such as essential oils (EOs), have been used in traditional medicine as anti-inflammatories, digestives, diuretics, expectorants and sedatives, along with other applications (Table 1). Nowadays, in addition to their use in aromatherapy, essential oils find application in cosmetics, cleaning products, fragrances, foods and beverages. Essential oils have been reported to show several biological properties, including antimicrobial, antioxidant, anti-inflammatory and anticancer properties, among others [10,11,12]. Particularly relevant is EOs’ anti-inflammatory activity, either by inhibiting several enzymes, such as oxygenases, nitric oxide synthases and peroxidases, or by inducing the release of pro-inflammatory cytokines, like interleukins and tumor necrosis factor-α (TNF‑α) [10]. 

Chemokines constitute a family of chemoattractant cytokines. These are small heparin-binding proteins involved in atherosclerosis by promoting directed migration of inflammatory cells. Chemokine (C-C motif) ligand 2 (CCL2), also known as monocyte chemoattractant protein-1 (MCP-1), has been detected in atherosclerotic lesions [34]. CCL2 is also a potent mediator of chronic inflammation, triggering, for instance, inflammation in rheumatoid arthritis [35]. In addition, inflammatory response is characterized by increased production of tumor necrosis factor-α (TNF-α) [35]. TNF-α, interleukin (IL)-1 and IL-6, secreted by macrophages, lymphocytes, natural killer cells and vascular smooth muscle cells, are considered pro-atherogenic cytokines [36]. Despite the reported anti-inflammatory potential of several EOs (Table 2), their effect on the release of CCL2 is much less reported than the release of TNF-α.

Essential oils are gaining commercial relevance in several countries, such as Portugal or Angola, as an additional source of income in a context of a more sustainable use of the local flora. Nevertheless, despite these essential oils being traded, national or internationally, for specific markets, it is ever more important for their added value to gather scientific support for their alleged biological properties. Given the traditional and commercial use of EOs for medicinal and cosmetic purposes and the knowledge of the ability, of their monoterpene and sesquiterpene constituents, to act as anti-inflammatories [56,57], the present work evaluated twenty EOs obtained from eighteen plant species collected in Portugal and Angola (Table 1) for their effect on the release of CCL2 (MCP‑1) and TNF-α by lipopolysaccharide (LPS)-stimulated THP-1 cells. 

## 2. Material and Methods

### 2.1. Plant Material

Collective and/or individual samples, from cultivated and wild-growing medicinal and aromatic plants, were collected from mainland Portugal (Table 3). As a rule, the plant material was collected during the local producers’ harvesting season. For herbaceous species, this was usually at the flowering phase, whereas for trees, it was at landscaping time. If not immediately extracted, the plant material was stored at −20 °C until essential oil (EO) isolation. Dried aerial parts from commercially available products sold in local herbal shops were also analyzed, as well as the essential oils isolated from oleoresin, in the case of *Pinus pinaster*, and from the two species from Angola (Table 3). A total of twenty essential oils isolated from eighteen species from the Amaranthaceae, Apiaceae, Asteraceae, Euphorbiaceae, Lamiaceae, Myrtaceae and Pinaceae families were tested. A voucher specimen of each plant species, collected from the wild state condition, was deposited in the Herbarium of the Botanical Garden of Lisbon University, Lisbon, Portugal. For commercial plant material, a reference sample from each plant is retained at the CBV laboratory and is available upon request.

### 2.2. Extraction and Chemical Analysis of the Essential Oils

Essential oils were extracted by hydrodistillation for 3 h, using a Clevenger-type apparatus, according to the European Pharmacopoeia [59], and stored at −20 °C until analysis. The EOs were analyzed by gas chromatography (GC) for component quantification and gas chromatography coupled to mass spectrometry (GC-MS) for component identification. 

#### 2.2.1. Gas Chromatography (GC)

Gas chromatographic analyses were performed using a Perkin Elmer Clarus 400 gas chromatograph equipped with two flame ionization detectors (FIDs), a data handling system and a vaporizing injector port into which two columns of different polarities were installed: a DB-1 fused-silica column (polydimethylsiloxane, 30 m × 0.25 mm i.d., film thickness 0.25 µm; J & W Scientific Inc., Rancho Cordova, CA, USA) and a DB-17HT fused-silica column ((50% phenyl)-methylpolysiloxane, 30 m × 0.25 mm i.d., film thickness 0.15 µm; J & W Scientific Inc.). The oven temperature was programmed from 45 to 175 °C, at 3 °C/min, and subsequently at 15 °C/min up to 300 °C, and then held isothermal for 10 min; injector and detector temperatures were 280 °C and 300 °C, respectively; the carrier gas, hydrogen, was adjusted to a linear velocity of 30 cm/s. The samples were injected using a split sampling technique, ratio 1:50. The volume of injection was 0.1 µL of *n*-pentane-essential oil solution (1:1). The percentage composition of the volatiles was computed, by the normalization method from the GC peak areas, and calculated as the mean values of two injections, from each sample, without using the response factors. 

#### 2.2.2. Gas Chromatography-Mass Spectrometry (GC‑MS)

The GC‑MS unit consisted of a Perkin Elmer Clarus 600 gas chromatograph, equipped with a DB‑1 fused-silica column (30 m × 0.25 mm i.d., film thickness 0.25 µm; J & W Scientific, Inc.), and interfaced with a Perkin Elmer 600T mass spectrometer (software version 5.4.2.1617, Perkin Elmer, Shelton, CT, USA). Injector and oven temperatures were as above; transfer line temperature, 280 °C; ion source temperature, 220 °C; the carrier gas, helium, was adjusted to a linear velocity of 30 cm/s; split ratio, 1:40; ionization energy, 70 eV; scan range, 40–300 u; scan time, 1 s. The identity of the components was assigned by comparison of their retention indices, relative to *n*‑alkane indices and GC‑MS spectra from a lab‑made library, created with reference essential oils, laboratory-synthesized components, laboratory-isolated compounds and commercially available standards.

### 2.3. In Vitro Inhibition of TNF-α and CCL2

This assay was performed according to Campana et al. [60]. Briefly, THP-1 cells (ATCC TIB-202) were cultivated in RPMI 1640 medium supplemented with 0.05 mM 2-mercaptoethanol, 10% fetal bovine serum (FBS), 100 U/mL penicillin and 100 μg/mL gentamicin at 37°C in an atmosphere containing 5% CO_2_. The medium was renewed twice a week when the cell concentration reached 1.0 × 10^6^ cells/mL. The cells were transferred to a 96-well microplate at a concentration of 100,000 cells per well and incubated for 18 h with RPMI supplemented with 1% FBS to initiate serum starvation, which was kept throughout the experiment. 

The cells were pre-treated with EOs at three concentrations for 3 h. To determine each EO working concentration, the toxicity of the EOs on THP-1 cells was accessed by measuring cell viability using a 3‑(4,5-dimethylthiazol-2-yl)-2,5‑diphenyltetrazolium bromide (MTT) method and untreated cells as the reference for viability [61]. EOs were considered nontoxic for the THP-1 cell line, and adequate for further analysis, when cell viability was higher than 90%. The EO concentrations ranged from 3 µg/mL to 90 µg/mL (*Dysphania ambrosioides*, *Eucalyptus globulus*, *E. radiata*, *E. viminalis*, *Foeniculum vulgare*, *Lavandula stoechas*, *Mentha cervina*, *Origanum majorana*, *Pinus pinaster*, *Solidago virgaurea, Thymus mastichina*, *Th. pulegioides* (*Thymus* abbreviated to *Th*., to avoid confusion with *T*. from *Thymbra*), *Th. vulgaris*), from 3 µg/mL to 30 µg/mL (*Brachylaena huillensis*, *Satureja montana*, *Spirostachys africanus*, *Th. zygis*) and from 5 µg/mL to 90 µg/mL (*Thymbra capitata*) (Table 4). 

Lipopolysaccharide (LPS) from *Escherichia coli* 0111:B4 (Sigma), added at 200 ng/mL, was employed as the inflammatory stimulus. The plate was incubated at 37°C overnight. After this period, the plate was centrifuged (1800 g, 5 min, 16°C), the supernatant collected and TNF-α release measured using the cytokine-specific sandwich quantitative ELISA according to the manufacturer’s instructions (TNF-α duo set, DY210, R&D Systems, Minneapolis, MN, USA). CCL2 release was measured using the cytokine-specific sandwich quantitative ELISA according to the manufacturer’s instructions (Human CCL2/MCP-1 duo set, DY279, R&D Systems, Minneapolis, MN, USA). Dexamethasone was employed as positive control (0.3 μM). The statistical significance of differences was calculated employing the software GraphPad Prism, version 5.0 (GraphPad Software Inc., San Diego, CA, USA), using ordinary one-way ANOVA/Newman–Keuls multiple comparison test. All the experiments were performed in triplicate.

## 3. Results and Discussion

### 3.1. Chemical Composition of the Essential Oils

All essential oils were fully chemically characterized. Table 3 reports only their main constituents (≥10%), since, in some cases, duly marked in Table 3, the detailed composition was previously reported, or their composition was overall very similar to data from prior studies. In the case of EOs from Angola, the complexity of the EOs still requires additional characterization for the full identification of some minor components.

Although commercialized as *Lavandula stoechas* L., the presence of necrodane derivatives, such as 5-methylene-2,3,4,4-tetramethylcyclopent-2-enone (18%) in the analyzed essential oil, undoubtedly indicated that the butterfly lavender tested was *L. luisieri* and not *L. stoechas* (Table 3). The chemical composition of *L. stoechas* essential oil is characterized by large variations in fenchone, camphor and 1,8‑cineole amounts, whereas necrodane derivatives are characteristic of *L. luisieri* [23,24,62]. Even though some variations in their contents were observed, the remaining essential oil compositions were in accordance with previous studies carried out with *Foeniculum vulgare* [15,63], *Mentha cervina* ([25] and references therein), *Origanum majorana* [64], *Satureja montana* [58,65], *Thymbra capitata*, *Thymus mastichina*, *Th. pulegioides*, *Th. vulgaris* and *Th. zygis* subsp. *zygis* [28,64,65,66], *Eucalyptus* species [67,68] and *Pinus pinaster* [33,69].

Although a few studies evaluated the EO composition from *Brachylaena huillensis* aerial parts, only three studies reported the essential oil composition from the wood or saw powder of this species [16,17,70]. Although no detailed composition has been reported, α‑amorphene was the dominant constituent in the studies of Klein and Schmidt [70] and of Maitai et al. [16] (17% and 15%, respectively), whereas β-caryophyllene (19%) was the major constituent described by Oliva et al. [17]. In the present study, α‑amorphene was the second main component, together with gleenol (both 6%), whereas β-caryophyllene was found only in trace amounts. Baarschers et al. [20] reported the isolation of diterpenes from *Spirostachys africana* wood, but no previous studies addressed the EO composition from the wood.

### 3.2. In Vitro Inhibition of TNF‑α Release by LPS-Stimulated THP-1 Cells

The potential anti-inflammatory activity of essential oils (EOs) was investigated by measuring TNF‑α release by lipopolysaccharide (LPS)-stimulated THP-1 cells by employing an immunoassay. The toxicity of the EOs on THP-1 cells was accessed to determine the adequate EO working concentrations. When the cell viability of THP-1 cells was higher than 90%, samples were considered non-cytotoxic and adequate for further analysis. According to the availability of EO, at least three concentrations were checked for each essential oil (Table 4). Data in Table 4 also include information on EOs which were not assessed further due to being toxic (ND) to differentiate them from those that showed no inhibition (NI).

The EOs of *T. capitata*, *L. luisieri*, *F. vulgare* and *D. ambrosioides* significantly reduced TNF-α release by LPS-stimulated THP-1 cells, in comparison to the control cells. From these four EOs, those of *T. capitata* and *L. luisieri* were the most effective. *T. capitata* EO showed an inhibition percentage of TNF-α release of 51 ± 7% at 20 μg/mL, whereas that of *L. luisieri* EO was 23 ± 1% at 30 μg/mL and 83 ± 8% at 90 μg/mL (Table 4). These inhibition percentages were higher than those of *D. ambrosioides* EO (49 ± 2% at 90 μg/mL), or *F. vulgare* EO (22 ± 2% at 90 μg/mL) (Table 4). 

The potential anti-inflammatory activity of *L. luisieri*, *F. vulgare* and *T. capitata* EOs has been previously reported using different in vitro and in vivo models (Table 2), but as far as we know, the anti-inflammatory potential of *D. ambrosioides* EO has not been addressed to date. Recently, the anti-inflammatory activity of alcoholic or hydroalcoholic extracts of *D. ambrosioides* was reported as showing the ability to reduce interleukin 6 (IL-6), myeloperoxidase (MPO), nitric oxide (NO) and adenosine-deaminase (ADA) activity and TNF-α and, therefore, they are potentially useful in wound healing and in the treatment of arthritic processes [13,71]. The oxygen-containing monoterpene ascaridole was identified as a constituent of *D. ambrosioides* ethanolic extract by Grassi et al. [13], a compound also identified in the essential oils evaluated in the present work (Table 3).

Despite carvacrol being the main compound of *T. capitata* EO (Table 3), this phenol-like oxygen-containing monoterpene may not be the only compound accountable for *T. capitata* EO activity. Indeed, other carvacrol-rich EOs, such as those of *S. montana* and *Th. zygis* (Table 3), were not able to reduce TNF‑α release. The presence of antagonists in these EOs can also not be ignored. Moreover, it is relevant to highlight the important role of the minor compounds and/or some of the compounds’ enantiomeric ratio in the overall activity of EOs. Often overlooked, these factors can contribute to synergistic or antagonistic actions determining differences in the EOs’ activities [56,72]. These results make it difficult to predict the effect of different species’ essential oils that share the same major component for TNF-α release. 

*Th. pulegioides* and, particularly, *Th. vulgaris* EOs, with thymol, an isomer of carvacrol as the main component (Table 3), were toxic for THP-1 cells, even at lower concentrations (Table 4). *Th. vulgaris* EO has been reported to show anti-inflammatory activity, including the capacity of reducing TNF-α release, this activity being related solely to the higher carvacrol content [37,49,50,52]. On the other hand, *Th. zygis* and *Th. vulgaris* EOs, which have thymol as the main constituent, have been reported to decrease TNF-α secretion by human macrophages derived from THP‑1 monocytes and activated by oxidized (ox)‑LDLs [51]. Dexamethasone at 0.3 μM had > 90% inhibition.

### 3.3. In Vitro Inhibition of CCL2 Release by LPS-Stimulated THP-1 Cells

Inflammatory changes in arterial lesions are characterized by the recruitment and activation of monocytes/macrophages, which are regulated by CCL2. This chemoattractant cytokine has been shown to play a vital role in the initiation and progression of arteriosclerotic lesions in experimental animals [73]. The effect of the essential oils on CCL2 release by LPS-stimulated THP‑1 cells was also evaluated.

Of the four essential oils with the ability to inhibit CCL2 release, only *L. luisieri* EO had remarkable activity, with inhibition percentages of 23 ± 1%, 54 ± 3% and 82 ± 12% at 10, 30 and 90 µg/mL, respectively (Table 4). The major compound of *L. luisieri* EO, 5-methylene-2,3,4,4-tetramethylcyclopent-2-enone, a necrodane derivative, may have contributed to this activity, along with 1,8-cineole. Nevertheless, the absence of the activity of other EOs in which 1,8-cineole was also present, even in a much higher percentage, such as *Th. mastichina* or *Eucalyptus* EOs (Table 3), may suggest that 5‑methylene-2,3,4,4-tetramethylcyclopent-2-enone plays an important role in the inhibition of both TNF‑α and CCL2 release (Table 4). The inhibitory activities elicited by *D. ambrosioides*, *S. virgaurea* or *B. huillensis* EOs were much lower (Table 4). Dexamethasone at 0.3 μM had > 90% inhibition.

Reports regarding the action of essential oils and/or their main components on the production of CCL2 are scarce. Limonene isolated from *Citrus junos* EO was able to inhibit CCL2 production on diesel exhaust particle (DEP)-stimulated human eosinophilic leukemia HL-60 clone 15 cells [74]. *Artemisia argyi* EO, mainly constituted by 1,8-cineole (33%), camphor (17%), (‑)‑borneol (13%) and α‑thujone (13%), reduced TNF-α, IL-6, IFN-β and CCL2 in LPS-induced RAW264.7 macrophages [75]. Xiao Qing Long Tang essential oil was able to suppress CCL2, IL-1β, IL-6, IL-10 and TNF-α expression and production by LPS-stimulated RAW264.7 cells [76]. In addition, Park et al. [77] also reported that (‑)‑linalool was able to inhibit microglial migration induced by CCL2, a chemokine released by oxygen-glucose deprivation/reoxygenation (OGD/R) in cortical cells from 17-day-old embryos of Sprague-Dawley rats.

Along with *L. luisieri* EO, the ascaridole- and *iso*-ascaridole-rich *D. ambrosioides* EO was also able to reduce CCL2 release by LPS-stimulated THP-1 cells, as observed for TNF-α, although in a weaker manner (Table 4). The absence of these compounds in the remaining non-active EOs may suggest that these volatile compounds have an important role in the suppression of some inflammatory processes in which TNF-α and CCL2 are involved. Despite the traditional application of *D. ambrosioides* as a vermifuge and against vomiting [14], this is the first report on the effect of its essential oil on the release of the pro-inflammatory cytokine TNF-α and the chemokine CCL2. For this reason, this EO and its main component ascaridole, and/or its isomers, should be further investigated to explore their anti-inflammatory activity.

## 4. Conclusions

Inflammatory disorders are usually treated with steroidal anti-inflammatory drugs (SAIDs) or non-SAIDs (NSAIDs). Nevertheless, because these drugs present multiple negative side effects, it is important to assess and validate the use of other potential anti-inflammatory agents, namely, essential oils. Moreover, some of these essential oils are by-products from landscaping activities or other industries, thus constituting an added value to countries’ local flora.

This study suggests that *T. capitata* and *L. luisieri* EOs, mainly constituted by carvacrol and 5‑methylene-2,3,4,4-tetramethylcyclopent-2-enone and 1,8-cineole, respectively, were the most effective to inhibit TNF‑α release by LPS-stimulated THP-1 cells, whereas only *L. luisieri* EO had the ability to inhibit CCL2 release by LPS-stimulated THP-1 cells. 

EOs’ chemical complexity and variability (existence of chemotypes and/or the enantiomeric ratio of some components), their hydrophobicity and, sometimes, their scarcity, have been considered some of the limitations to their use in diverse formulations. Nevertheless, EOs are Generally Regarded as Safe (GRAS), and the knowledge on their biological properties should be further explored, in solo formulations and in combination therapies, as potential anti-inflammatory agents. This approach would contribute to the goal of decreasing the use of SAIDs and, therefore, preventing or diminishing these drugs’ adverse side effects.

## Figures and Tables

**Table 1:** Some of the traditional applications of the species studied in the present work.

Family/Plant Species	Common Names (pt/en)	Medicinal Use	Other Uses	Reference
**Amaranthaceae**				
*Dysphania ambrosioides* (L.) Mosyakin & Clemants (= *Chenopodium ambrosioides* L.)	Quenopódio/Wormseed	Against respiratory, gastrointestinal and joint inflammatory disorders	Vermifuge, emetic	[13,14]
**Apiaceae/Umbelliferae**				
*Foeniculum vulgare* Mill.	Funcho/Fennel	Against respiratory and gastrointestinal inflammatory disorders	Culinary (seasoning)	[15]
**Asteraceae/Compositae**				
*Brachylaena huillensis* O. Hoffm. (= *Brachylaena hutchinsii* Hutch., *Brachylaena mullensis* O.Hoffm.)	Muhuhu */Silver oak	Against schistosomiasis and roots against diabetes	Firewood, charcoal, timber, poles, posts, tool handles, carving. Perfumery (essential oil distilled from wood)	[16,17,18]
*Solidago virgaurea* L.	Vara-de-ouro/European goldenrod or woundwort	External and internal against urinary inflammatory disorders	Cosmetic	[19]
**Euphorbiaceae**				
*Spirostachys africana* Sond. [= *Excoecaria africana* (Sond.) Müll.Arg., *Excoecaria synandra* Pax, *Excoecariopsis synandra* (Pax) Pa, *Sapium africanum* (Sond.) Kuntze, *Spirostachys synandra* (Pax) Pax, *Stillingia africana* (Sond.) Baill.]	Tambooti **	External to treat myiasis, internal against gastrointestinal inflammatory disorders	Use of wood in furniture	[20,21,22]
**Lamiaceae/Labiatae**				
*Lavandula luisieri* (Rozeira) Rivas-Martínez	Rosmaninho/butterfly lavender	External and internal against respiratory, circulatory, gastrointestinal and joint inflammatory disorders	Ornamental, aromatic, cosmetic, culinary (seasoning)	[23,24]
*Mentha cervina* L.	Poejo fino/Hart’s pennyroyal	External and internal against respiratory and gastrointestinal inflammatory disorders	Aromatic, culinary (seasoning)	[25]
*Origanum majorana* L.	Oregão/Marjoram	External and internal against nervous, respiratory and gastrointestinal inflammatory disorders	Aromatic, culinary (seasoning)	[26]
*Satureja montana* L.	Segurelha/Winter savory	External and internal against nervous, respiratory and gastrointestinal inflammatory disorders	Culinary (seasoning)	[27]
*Thymbra capitata* (L.) Cav. [= *Thymus capitatus* Hoffms. et Link., *Thymus creticus* Brot., *Corydothymus capitatus* Rechenb. f., *Satureja capitata* L.]	Tomilho-de-Creta/Conehead thyme	External and internal against spasms and nervous, respiratory and gastrointestinal disorders	Aromatic, culinary (seasoning)	[28]
*Thymus mastichina* (L.) L.	Bela-luz/Spanish marjoram	External and internal against nervous, respiratory, gastrointestinal and joint inflammatory disorders	Aromatic, culinary (seasoning)	[28]
*Thymus pulegioides* L.	Serpão/Broad-leaved thyme, lemon thyme	External and internal against nervous, respiratory and gastrointestinal inflammatory disorders	Aromatic, culinary (seasoning)	[29]
*Thymus vulgaris* L.	Tomilho/thyme	External and internal against nervous, respiratory and gastrointestinal inflammatory disorders	Ornamental, aromatic, culinary (seasoning)	[19]
*Thymus zygis* Loefl. ex L. subsp. *zygis*	Erva-de-Santa-Maria/Spanish red thyme	External and internal against nervous, circulatory, respiratory and gastrointestinal inflammatory disorders	Aromatic, culinary (seasoning)	[28]
**Myrtaceae**				
*Eucalyptus globulus* subsp. *maidenii* (F.Muell.) J.B.Kirkp.	Eucalipto/Maiden’s gum	External and internal against circulatory, respiratory and gastrointestinal inflammatory disorders	Timber, fuel, paper pulp. Aromatic, culinary (seasoning)	[30]
*Eucalyptus radiata* A.Cunn. ex DC.	Eucalipto/Narrow-leaved peppermint eucalyptus	External and internal against mouth, respiratory and gastrointestinal inflammatory disorders		[31]
*Eucalyptus viminalis* Labill.	Eucalipto/Manna gum	Internal against respiratory inflammatory disorders	Deodorant	[32]
**Pinaceae**				
*Pinus pinaster* Aiton	Pinheiro-bravo/Maritime pine	External for circulatory problems, and internal against respiratory, gastrointestinal and joint inflammatory disorders	Timber and oleoresin production	[33]

pt/en: Official two-letter codes of Portuguese and English languages, respectively. * African name adopted in Portuguese. ** African name given to the wood and adopted in Portuguese and English.

**Table 2:** Previously reported anti-inflammatory activity of the essential oils (EOs) from the species under study.

EO/EO Components’ Anti-Inflammatory Activity	Family/Species	Reference
**Apiaceae**		
*Foeniculum vulgare*	EO inhibition of 5-lipoxygenase (IC_50_ = 0.04 mg/mL). Fenchone inhibition of 5-lipoxygenase (IC_50_ = 0.02 mg/mL).	[37]
	EO (200 and 400 mg/kg) decreased the activity of mieloperoxidase (MPO) and the expression of TNF-α in the colon tissue previously submitted to acetic acid solution (acute colitis), and inhibited acetic acid-induced expression of p‑NF-kB p65 protein.	[38]
**Lamiaceae**		
*Lavandula luisieri*	EO (50–200 mg/kg) inhibition of paw edema (31–83%) induced by carrageenan administered in male Wistar rats.	[39]
	EO (25 μg/mL) nitric oxide (NO) inhibition (75%) in IL-1β induced primary chondrocyte.	[40]
	EO reduction of iNOS in human chondrocytes and intestinal cell line C2BBe1 (54.9 and 81.0%, respectively) and phosphorylated IkB-α (87.4% and 62.3%, respectively).	[41]
*Origanum majorana*	EO (10 μg/mL) diminished the TNF-α, IL‑1β, IL-6, IL-10 and COX-2 secretion and NFκB gene expression after activation of THP-1 cells by lipopolysaccharide or human ox – LDL. The activity was attributed to *cis*-sabinene hydrate and terpinen‑4‑ol.	[42]
*Thymbra capitata*	EO inhibition of 5-lipoxygenase (IC_50_ = 0.1 mg/mL).	[43]
	EO inhibition of 5-lipoxygenase (IC_50_ = 0.2 mg/mL).	[44]
*Thymus mastichina*	EO inhibition of 5-lipoxygenase (IC_50_ = 0.7 mg/mL).	[43]
*Thymus vulgaris*	EO inhibition of 5-lipoxygenase (IC_50_ = 0.19 μg/mL).	[37]
	EO (0.5 μg/mL) inhibition (80%) of 5-lipoxygenase.	[45]
	EO inhibition of 5-lipoxygenase (IC_50_ = 0.005 μg/mL). EO reduced the TNF-α, IL-1β, IL-8 secretion levels of THP-1 cells.	[46]
	EO (400 mg/kg, after 6 h) reduced (50.4–58.4%) carrageenan-induced paw edema in mice.	[47]
	Carvacrol (10 mg/ear) reduced ear edema. Carvacrol (10 mg/ear) inhibited the activity of myeloperoxidase (MPO) (43.8%). Carvacrol (0.3–90 μg/mL) reduced (20.07–52.23%) neutrophil migration in response to fMLP stimulation. EO (750 mg/kg) and carvacrol (100–400 mg/kg) exerted inhibited leukocyte migration to the injury site. Carvacrol (0.3‑90μg/mL) reduced LTB4 stimulation (19.8–61.1%).	[48]
	EO and carvacrol suppressed lipopolysaccharide-induced COX-2 mRNA and protein expression in human macrophage-like U937 cells.	[49]
	EO (moderate concentration) decreased the mRNA levels of IL-1β, IL-6, granulocyte-macrophage colony stimulating factor (GM-CSF) and TNF-α, and lowered the amount of IL-1β and IL-6 proteins in animal models of colitis.	[50]
	EO reduced production and gene expression of the pro-inflammatory mediators TNF‑α, IL-1B and IL-6 and increased the parameters on the anti-inflammatory IL-10 cytokine.	[51]
	EO (5000 ppm) decreased paw edema and ear swelling, inhibited the total mRNA IL‑1β expression in the mouse colon.	[52]
*Thymus zygis* subsp. *zygis*	EO thymol type inhibition of 5-lipoxygenase (IC_50_ = 54 – 73 μL/L).EO linalool type inhibition of 5-lipoxygenase (IC_50_ = 299 – 402 μL/L).	[53]
	EO reduced production and gene expression of the pro-inflammatory mediators TNF‑α, IL-1B and IL-6 and increased the parameters on the anti-inflammatory IL-10 cytokine.	[51]
**Myrtaceae**		
*Eucalyptus globulus*	EO inhibition of 5-lipoxygenase (IC_50_ = 0.16 mg/mL).	[37]
subsp. *maidenii*	EO (0.5μg/mL) inhibition (50%) of lipoxygenase.	[45]
	EO (200 mg/kg) inhibited by 28.8% the inflammatory phase of wound healing (Whittle method).	[54]
**Pinaceae**		
*Pinus pinaster*	EO (100 mg/kg dose) inhibition (30.3%) of paw edema in the Whittle method using carrageenan.	[55]

EO: Essential oil. IC_50_: Half-maximal inhibitory concentration. LDL: Low-density lipoprotein. COX‑2: Ciclo‑oxigenase-2.

**Table 3:** List of the evaluated species, sampling year, plant part used for hydrodistillation, plant source, essential oil yield and main components (≥10%).

Family/Species	Code	Sampling Date	Plant Part	Collection Place/Source ^#^	EO yield (%, *v*/*w*)	Main Components (≥10%)
**Amaranthaceae**						
*Dysphania ambrosioides*	Da	2013	FF	Monsaraz	0.56	*iso-*Ascaridole 51, ascaridole 16
						
**Apiaceae**						
*Foeniculum vulgare*	Fv	2013	DV	Herbal shop		α-Pinene 27, *trans*-anethole 18, Limonene 11
	Fv s	2013	Seeds	Herbal shop	1.16	Methyl chavicol 79, limonene 12
**Asteraceae**						
*Brachylaena huillensis*	Bh	2013	EO	Angola	n.a.	Copaen-15-ol * 14
	Bh *	2013	EO*	Angola	n.a.	Copaen-15-ol * 12
*Solidago virgaurea* ^a^	Sv	2013	FF	Pinheiro da Cruz	0.72	β-Pinene 22, α-pinene 21, germacrene D 15, limonene 12
**Euphorbiaceae**						
*Spirostachys africana*		2013	EO	Angola	n.a.	Stachenone * 28, Diosphenol (2) * 38
**Lamiaceae**						
*Lavandula luisieri* ^a,b^	Ll	2013	DF	Herbal shop	0.44	5-Methylene-2,3,4,4-tetramethylcyclopent-2-enone 18, 1,8-cineole 16
*Mentha cervina*	Mc	2013	DV	Herbal shop	1.54	Pulegone 76
*Origanum majorana* ^a^	Om	2013	DV	Herbal shop	0.98	Terpinen-4-ol 18, carvacrol 17, γ‑terpinene 13, carvacrol methyl ether 13
*Satureja montana* ^a^	Sm	2013	DV	Herbal shop	1.48	Carvacrol 77
*Thymbra capitata*	Tc	2013	FV	Algarve	0.89	Carvacrol 71
*Thymus mastichina*	Thm	2013	FF	Bragança	1.35	1,8-Cineole 69
*Thymus pulegioides* ^a^	Thp	2013	DL	Herbal shop	0.49	Thymol 32, *ρ*-cymene 22
*Thymus vulgaris* ^a^	Thv	2013	DV	Herbal shop	1.20	Thymol 45, *ρ*-cymene 21, γ‑terpinene 16
*Thymus zygis* subsp. *zygis* ^a^	Thzz	2013	FF	Bragança	0.71	Carvacrol 45, *ρ*-cymeme 22, γ‑terpinene 17
**Myrtaceae**						
*Eucalyptus globulus* subsp. *maidenii*	Eg	2013	FL	MEE	3.20	α-Pinene 15, 1,8-Cineole 46, Limonene 23
*Eucalyptus radiata*	Er	2012	FL	MEE	7.20	1,8-Cineole 49
*Eucalyptus viminalis*	Ev	2012	FL	MEE	2.50	α-Pinene 10, 1,8-Cineole 69
**Pinaceae**						
*Pinus pinaster*	Pp	2013	Oleoresin	Nazaré	29.76	α-Pinene 62, β-pinene 23

^#^ Unless otherwise specified, the collection place was in Portugal. ^a^ Detailed composition of EOs reported in Faria et al. [58]. ^b^ Commercialized as *Lavandula stoechas* L. n.a.: Information not available. * Identification based on mass spectra only. DF: Dry, flowering phase aerial parts. DL: Dry leaves. DV: Dry, vegetative phase aerial parts. EO: Essential oil supplied by the producer, obtained from the wood. EO*: In-lab re-distilled essential oil supplied by the producer, due to some turbidity of the original sample. FF: Fresh, flowering phase aerial parts. FL: Fresh leaves from fruiting phase. MEE: Mata Experimental do Escaroupim.

**Table 4:** Inhibition of TNF-α and CCL2 production by lipopolysaccharide (LPS)-activated THP-1 monocytic cells elicited by the evaluated essential oils (EOs).

Family/Species and Control	Concentrations (µg/mL)	TNF-α Inhibition (% ± S.D., *n* = 3)	CCL2 Inhibition (% ± S.D., *n* = 3)
**Control**	LPS (200 ng)	2428.1 ± 587.8 ^a^	2382.3 ± 1480.8 ^a^
	DMSO (0.1%)	96.5 ± 13.9 ^a^	24.1 ± 14.7 ^a^
**Amaranthaceae**			
*Dysphania ambrosioides*	90	48.6 ± 2.1 ***	15.6 ± 0.7 ***
	30	30.9 ± 1.5 *	9.4 ± 0.3 **
	10	13.6 ± 1.0	7.5 ± 0.3 *
**Apiaceae**			
*Foeniculum vulgare*	90	22.3 ± 1.9 ***	NI
	30	0.5 ± 0.0	NI
	10	NI	NI
**Asteraceae**			
*Brachylaena huillensis*	30	NI	18.8 ± 2.3 **
(re-distilled EO)	10	4.4 ± 0.8	9.0 ± 0.5
	3	NI	5.4 ± 0.2
*Brachylaena huillensis*	30	ND	ND
	10	ND	ND
	3	ND	ND
*Solidago virgaurea*	90	ND	ND
	30	5.0 ± 0.2	ND
	10	NI	4.9 ± 0.1 *
	3	ND	8.0 ± 0.1 **
**Euphorbiaceae**			
*Spirostachys africanus*	30	ND	ND
	10	ND	ND
	3	ND	ND
**Lamiaceae**			
*Lavandula luisieri*	90	82.9 ± 8.2 ***	82.0 ± 12.4 ***
	30	23.2 ± 1.1 ***	54.3 ± 3.0 ***
	10	2.5 ± 0.1	22.7 ± 1.0 ***
*Mentha cervina*	90	NI	NI
	30	NI	NI
	10	NI	2.5 ± 0.0
*Origanum majonara*	90	NI	NI
	30	NI	NI
	10	NI	4.8 ± 0.1
*Satureja montana*	30	ND	ND
	10	ND	ND
	3	NI	0.2 ± 0.0
*Thymbra capitata*	30	ND	NI
	20	51.1 ± 6.5 ***	ND
	10	29.5 ± 1.7 ***	0.4 ± 0.0
	5	9.1 ± 0.1 *	ND
*Thymus mastichina*	90	ND	ND
	30	ND	ND
	10	ND	ND
*Thymus pulegioides*	90	ND	ND
	30	NI	0.9 ± 0.0
	10	NI	8.4 ± 0.5
*Thymus vulgaris*	90	ND	ND
	30	ND	ND
	10	ND	ND
*Thymus zygis* ssp. *sygis*	30	NI	8.9 ± 1.0
	10	NI	2.7 ± 0.2
	3	NI	0
**Myrtaceae**			
*Eucapyptus globulus* subsp.	90	6.5 ± 0.4	1.7 ± 0.0
*maidenii*	30	4.4 ± 0.3	NI
	10	2.4 ± 0.2	NI
*Eucalyptus radiata*	90	12.0 ± 0.1 *	NI
	30	NI	NI
	10	NI	NI
*Eucalyptus viminalis*	90	3.3 ± 0.2	NI
	30	0.2 ± 0.0	NI
	10	NI	1.4 ± 0.0
**Pinaceae**			
*Pinus pinaster* (oleoresin)	90	ND	ND
	30	6.3 ± 0.1	NI
	10	6.8 ± 0.3	NI

^a^ Inflammatory mediator production (absolute values in pg/mL). NI: No inhibition. ND: Not determined due to toxicity (cell viability ≤ 90%). * *p* ≤ 0.05; ** *p* ≤ 0.01; *** *p* ≤ 0.001: Indicates significant inhibition of TNF-α or CCL2 release in comparison to LPS-stimulated cells (ordinary one-way ANOVA/Newman–Keuls multiple comparison test: GraphPad Prism).

## Data Availability

Not applicable.

## Convention on Biodiversity

The authors obtained, and acknowledge, the appropriate authority to access plant samples, other than commercially available plant material, essential oils or oleoresin, used for research as required under the framework of the United Nations Convention on Biodiversity.

## Abbreviations

CCL2	Chemokine (C-C motif) ligand 2
COX-2	Cyclooxygenase-2
DF	Dry, flowering phase aerial parts
DL	Dry leaves
DV	Dry, vegetative phase aerial parts
EOs	Essential oils
FBS	Fetal bovine serum
FF	Fresh, flowering phase aerial parts
FL	Fresh leaves from fruiting phase
IC_50_	Half-maximal inhibitory concentration
LDL	Low-density lipoprotein
LPS	Lipopolysaccharide
MCP-1	Monocyte chemoattractant protein-1
MEE	Mata Experimental do Escaroupim
MTT	3-(4,5-Dimethylthiazol-2-yl)-2,5 diphenyltetrazolium bromide
THP-1	Human acute monocytic leukemia cell line
TNF-α	Tumor necrosis factor-α
